# Predisposing factors influencing occupational injury among frontline building construction workers in Ghana

**DOI:** 10.1186/s13104-019-4744-8

**Published:** 2019-11-06

**Authors:** John Amissah, Eric Badu, Peter Agyei-Baffour, Emmanuel Kweku Nakua, Isaac Mensah

**Affiliations:** 1 Department of Health Policy, Management and Economics, School of Public Health, Kwame Nkrumah University of Science and Technology, Kumasi, Ghana; 2 Department of Epidemiology and Biostatics, School of Public Health, Kwame Nkrumah University of Science and Technology, Kumasi, Ghana; 3 School of Nursing and Midwifery, University of Newcastle, Callaghan, Australia; 4 Isaac Mensah, Department of Special Education, University of Education, Winneaba, Ghana

**Keywords:** Occupational Injury, Kumasi, Ghana, Prevalence, Frontline construction workers

## Abstract

**Objective:**

This study aims to examine the predisposing factors influencing occupational injuries among frontline construction workers in Ghana. A cross-sectional survey was carried out with 634 frontline construction workers in Kumasi metropolis of Ghana using a structured questionnaire. The study was conducted from December 2016 to June 2017 using a household-based approach. The respondents were selected through a two-stage sampling approach. A multivariate logistics regression model was employed to examine the association between risk factors and injury. Data was analyzed employing descriptive and inferential statistics with STATA version 14.

**Results:**

The study found an injury prevalence of 57.91% among the workers. Open Wounds (37.29%) and fractures (6.78%) were the common and least injuries recorded respectively. The proximal factors (age, sex of worker, income) and distal factors (e.g. work structure, trade specialization, working hours, job/task location, and monthly off days) were risk factors for occupational injuries among frontline construction workers. The study recommends that policymakers and occupational health experts should incorporate the proximal and distal factors in the design of injury prevention as well as management strategies.

## Introduction

An occupational injury is described as any personal injury, disease or death that results from an occupational accident [1]. Globally, occupational injury has been identified as the leading cause of industrial ailment accounting for over 11% of disability [2–4]. Consistently, estimates suggest that over 350,000 people globally suffer from workplace injuries [5, 6]. Reporting information on occupational injuries is essential to assess the extent to which workers are protected from work-related hazards and risks [7].

Several studies and theories have been employed to explain the factors that influence occupational injuries. Generally, the constrain-response accident model has widely been used in the construction literature [8]. The theory suggests that each individual at the workplace plays a unique role and in the course of executing those roles is constrained by certain factors and their response to these constrain also create an additional set of constrain for other participants who depend on the formal’s actions to act [8, 9]. In particular, occupational injury is seen as a product of the interaction between management, organizational and operational features. This theory is explained according to two main constructs, namely proximal and distal factors [8, 9]. It argued that certain deficiencies in institutional activities could trigger an employee’s action that could lead directly or indirectly to an occurrence of an accident and eventually to an injury [10–17]. Those deficient factors that directly increase an individual’s risk to an accident are known as the proximal factors. These proximal factors do not predict that occupational injury will definitely happen, but rather indicates that a worker may be at risk of occupational injury at some time in the future [8, 9].

The distal factors, on the other hand, are managerial or organizational constrain experienced by employees and their responses to those constraints. They are those factors that could lead, with inappropriate response/action from employees indirectly to increasing the risk of accident causation as a result of the presence of the proximal factors [18]. The distal variables highlight organizational factors (e.g., management, organization and operation) that makes the individual vulnerable for an occupational injury. The distal factors which can increase the risk of occupational injuries are overtime measures, production target, number of working hours, type of work structure, income and task location [8–17]. Conversely, the proximal factors may include immediate individual lifestyle characteristics (e.g. alcohol consumption, smoking, adherence to safety regulation, type of work and exposure to hazards) and socio-demographic profile (e.g. age, gender, work experience) [13, 19]. More specifically, the distal factors lead to the introduction of proximal factors, whereas the proximal factors lead to the accident causation with injury as one of the final outcomes [11, 14].

In Ghana, occupational injuries have been identified among the leading causes of death. Specifically, 56 out of 902 occupational accidents reported in the year 2000 were from construction-related injuries [20]. This calls for government policies and interventions regarding occupational injuries in the construction industry. Despite this, little is known about the predictive factors that contribute to the burden of occupational injuries among construction workers. The few empirical studies measuring the prevalence and risk factors on injuries are limited to mining [21], domestic setting [3], transportation and manufacturing industries [22, 23], with little or no attention on building construction [17, 24, 25]. To our knowledge, no study has measured factors influencing occupational injuries among construction workers. Therefore, this paper aims to contribute to this gap by exploring the prevalence and predisposing factors influencing occupational injuries among frontline construction workers in Ghana.

## Main text

### Methodology

A cross-sectional design was employed to collect data from frontline building construction workers from December 2016 to June 2017. The study was limited to building construction workers in the Kumasi Metropolis of Ghana. Kumasi metropolis was selected due to its cosmopolitan nature, heterogeneous population and economic activities with distinct cultural enclaves making it a representation of the behaviour of building construction workers in Ghana [26]. The study recruited workers aged between 15 and 64 years who were actively engaged in construction activities for at least 12 calendar months. The workers were limited to carpenters, masons, brick/block layers, steel benders, and laborers. A sample size of 635 workers was estimated using Cochrane formulae [27, 28]. An assumed prevalence of 50% injuries in the construction industry with a precision of 5%, a design effect of 1.5% and non-response of 10% were used to estimate the sample size. A multi-stage sampling method was used to select respondents. The first stage involved selecting ten sub-metro in Kumasi metropolis. A simple random sampling was employed to select participants from households in the communities located in each sub-metro.

#### Data collection

Data were obtained through the administration of structured questionnaires on a face-to-face basis. The variables used in the questionnaire were identified from previous literature, theories, and standard injury questionnaire. A questionnaire template was developed and loaded onto a smartphone with ODK (Open Data kit) [29, 30]. The interviews were conducted in the respondent’s preferred dialect (e.g. mostly Asante Twi) [22]. Research assistants were trained on the use of ODK data collection software. Written informed consent was obtained from all participants enrolled in this study and identities of participants were kept anonymous. Consent was also sought from caretakers and parents of minor workers below 18 years who were actively involved in construction work. The questionnaire was pre-tested at Ejisu-Juaben Municipality, to check its reliability. The weakness identified was effected on the final questionnaire.

#### Data analysis

The data were summarized using descriptive and inferential statistics. Multivariate Logistics regression analysis was employed to identify the predisposing factors influencing occupational injuries. The injury was operationalized as all physical harm or damage to a person’s body caused by an object [1, 15]. The dependent variable was an injury sustained by a worker and the independent variables were individual factors such as demographic, socio-economic, job conditions, alcohol consumption, overtime and rest time. Data were analyzed using STATA 14, the significant level was maintained at a *P* value ≤ 0.05 and presented at 95% confidence interval. All monetary values were converted from the Ghanaian cedis (GHS) to the United States dollar (US$) equivalence using the prevailing exchange rate of GHS 4.2765: 1 US $ as published central bank of Ghana [31].

### Results

The mean age of the workers was 31.43 ± 8.9 years, the majority were males (89.3%) and married (58.8%). About 53.3% had basic level education and 22.5% had no formal education. The years of working experience were 7.57 ± 7.4. About 44.4% worked as daily paid workers, 17.6% as permanent and 37.9% as temporal workers. More than half were labourers (56.2%). Fifty percent earned a monthly income of GHS 960.0 (US $ = 224.5) (Table 1).

**Table 1:** Demographic characteristics

Characteristics	Frequency (n = 634)	Percentage
Age group		
15–24 years	143	22.5
25–34 years	298	46.8
35–44 years	136	21.5
45 + year	57	9.3
Mean (SD)	31.43 (8.85)	
Sex		
Male	569	90.0
Female	63	9.9
Marital status		
Single	262	41.1
Married	375	58.8
Educational level		
No Formal education	143	22.5
Basic education^a^	341	53.3
High school	137	21.5
Tertiary^b^	16	2.5
Working experience (years)		
1–4 years	304	47.7
5–9 years	143	22.5
10–20 years	157	24.6
21+	33	5.2
Mean SD	7.57 (7.40)	
Work structure		
Daily paid worker (by day work)	283	44.4
Temporal workers	242	37.9
Permanent worker	111	17.6
Monthly income in GHS [US$]		
360–499 [84.2–116.7]	19	2.9
500–999 [116.9–233.6]	389	61.1
1000–1499 [233.8–350.5]	204	32.0
1500 + [350.8]	25	3.9
Average income in GHS (SD)	954.86 [223.3] ± *311.89 [72.9]*	
Median [US $]	960 [224.5]	
Average wage [US $] ±* SD* per Artisan	Median [US$] wage per Artisan	
Mason	60 [14.0] ± *22.8 [5.3]*	50 [11.7]
Labourer	33.70 [7.9] ± *6.3 [1.5]*	30 [7.0]
Carpenter	52.86 [12.4] ± *12.9 [3.0*]	60 [14.0]
Steel bender	46.31 [10.8] ± *10.8 [2.5]*	50 [11.7]
Average wage per artisans [US $] ±* SD*	40.65 [9.5] ± *13.0 [3.0]*	
Trade specialization		
Mason	176	27.6
Labourer	358	56.2
Carpenter	70	10.9
Steel bender	33	5.1

GHS 4.2765: US$ 1

^a^Basic education: nine-year training from primary one to completion junior high school and Middle School

^b^Tertiary education: includes University and Polytechnics

#### The prevalence and types of occupational injuries

The injury prevalence among the frontline construction workers was 57.9%. About 37.3% experienced open wound, superficial (on surface) (15.3%), concussion and internal injuries (15.3%), dislocation and sprains (10.5%), traumatic amputation and deformity (8.5%), fractures (6.8%) and 6.5% suffered from other injuries (Table 2).

**Table 2:** Body regions, injury types and frequencies

Variable	Frequency	Percentage
Injury experience in last 12 months		
Yes	355	57.9
No	258	42.0
Type of injury		
Fracture	24	6.7
Open wound	132	37.3
Dislocation and sprains	37	10.5
Superficial (on surface) injury	54	15.3
Concussion and internal injury	54	15.3
Traumatic amputation and deformity	30	8.5
Other specific injuries^a^	23	6.5

^a^Other specific Injuries: All those including effects of heat, electric shocks, physical and psychological abuses, multiple injuries without specific description

#### The predictors of occupational injuries

In the multivariate analysis, covariates such as age, sex, type of work structure, income, trade specialization, working hours, job location and monthly off-days were statistically significant predictors of injury (Table 3).

**Table 3:** Univariate and multivariate analysis of risk factors for occupational injuries among frontline building construction workers in Ghana, 2016/17

Variables	Univariate model		Multivariate model	
	Crude odd ratio (OR; 95% CI)	P-value	Adjusted odd ratio (aOR; 95% CI)	P-value
Age group				
15–24 years (ref)	1.00	–	1.00	–
25–34 years	0.77 (0.51, 1.16)	0.21	0.41 (0.20, 0.80)	*0.009*
35–44 years	1.04 (0.64, 1.69)	0.88	0.53 (0.19, 1.44)	0.21
45+ year	2.48 (1.20, 5.13)	*0.01*	0.78 (0.23, 2.63)	0.68
Sex of worker				
Female (ref)	1.00	–	1.00	–
Male	5.51 (2.90, 10.45)	*0.001*	6.07 (2.41, 15.29)	*0.001*
Marital status				
Single (ref)	1.00	–	–	–
Married	1.25 (0.91, 1.73)	0.18	–	–
Educational level				
No formal education ref	1.00	–	–	–
Basic education	0.65 (0.42, 0.99)	*0.04*	–	–
High school	0.61 (0.36, 1.03)	0.06	–	–
Tertiary	0.75 (0.27, 2.14)	0.60	–	–
Working experience (years)				
1–4 years	0.23 (0.09, 0.56)	*0.001*	–	–
5–9 years	0.41 (0.16, 1.07)	0.068	–	–
10–20 years	0.34 (0.13, 0.87)	*0.02*	–	–
21+ (Ref)	1.00	–	–	–
Income				
360–499 [84.2–116.7] (ref)	1	–	1	–
500–999 [116.9–233.6]	1.28 (0.50, 3.24)	0.610	1.37 (0.38, 4.92)	0.62
1000–1499 [233.8–350.5]	3.79 (1.45, 9.92)	*0.007*	3.52 (0.98, 12.64)	*0.05*
1500 + [350.8]	30.25 (3.35, 273)	*0.002*	22.63 (1.60, 320.51)	*0.02*
Work structure				
Daily paid worker (by day work) (Ref)	1.00	–	1.00	–
Temporal contract workers	1.24 (0.87, 1.76)	0.231	2.03 (1.25, 3.31)	*0.004*
Permanent worker	4.60 (2.68, 7.89)	*0.001*	3.97 (1.93, 8.17)	*0.001*
Trade specialization				
Mason (ref)	1	–	1	–
Labourer	1.85 (1.27, 2.69)	*0.001*	0.77 (0.37, 1.57)	0.46
Carpenter	0.46 (0.24, 0.89)	*0.02*	1.09 (0.40, 3.00)	0.85
Steel bender	1.33 (0.61, 2.89)	0.47	0.22 (0.07, 0.64)	*0.01*
Working hours				
4–6 h	0.12 (0.05, 0.25)	*0.001*	0.05 (0.02, 0.15)	*0.001*
7–9 h	0.23 (0.09, 0.63)	*0.004*	0.16 (0.04, 0.36)	*0.009*
10+ h (ref)	1	–	1	–
Safety instruction				
No	1.27 (0.82, 1.99)	0.27	–	–
Yes (ref)	1	–	–	–
Currently smoking				
Never smoked (ref)	1.00	–	–	–
Currently smokes	2.41 (0.78, 7.49)	0.13	–	–
Alcohol consumption				
Do not consume (ref)	1.00	–	–	–
Consume	1.69 (1.13, 2.55)	*0.010*	–	–
Job/task location				
Rooftop	1	–		–
Lower floor/ground	0.22 (0.09, 0.48)	*0.001*	0.61 (0.16, 2.31)	0.46
Height from ground	0.19 (0.08, 0.45)	*0.001*	0.82 (0.21, 3.26)	0.78
All location	0.18 (0.80, 0.38)	*0.001*	0.29 (0.85, 1.01)	*0.05*
Daily production target				
No	1	–	–	–
Yes	0.75 (0.53, 1.05)	*0.100*	–	–
Monthly off days				
No	1	–	–	–
Yes	0.20 (0.11, 0.38)	*0.001*	*0.96 (0.04, 0.24)*	*0.001*
Monthly working days				
20 days	1	–	–	–
24 days	0.86 (0.54, 1.34)	0.496	–	–
26 days	0.48 (0.15, 1.49)	0.203	–	–
30 days	2.55 (0.51, 12.68)	0.252	–	–

Significant values are in italics

Workers between ages 25–34 were [aOR = 0.41; 95% CI = 0.20, 0.80] less likely to be exposed to the risk of injury than those below 24 years. The male workers were more likely to experience injury [aOR = 6.07; 95% CI = 2.41–15.29] compared to females. Consistently, workers who engaged permanently [aOR = 3.97; 95% CI. = 1.93–8.17] and temporal basis [OR = 2.03; 95% CI. = 1.25–3.31] were more likely to experience an injury than those working on daily basis. High income workers above GHS 1000 (US $ 233.87) [aOR = 3.52; 95% CI. = 0.98–12.64] and GHS 1500 (US $ 350.83) [aOR = 22.63; 95% CI. = 1.60–320.51] were more likely to be exposed to injury than low income workers. Steel Bender/Fixers were 78% protected from injury [aOR = 0.22; 95% CI = 0.07–0.64] compared with other trade specialization. Similarly, Risk was reduced by 95% [aOR = 0.05; 95% CI = 0.02–0.15] and 84% [aOR = 0.16; 95% CI = 0.04–0.36] for workers working between 4–6 h and 7–9 h daily compared to those working above 10 h. Also, workers operating from all location at the workplace could avert injury by 71% [aOR = 0.29; 95% CI = 0.85–1.01] compared to working on rooftops and from height. Workers who were entitled to off-days were 96% protected from the possibility of experiencing injury [aOR = 0.96; 95% CI = 0.04–0.24] compared to those without off-days.

### Discussion

The study adds to the existing literature, as the findings are discussed using the constraint-response accident model. The constructs used to organize the discussion are (1) prevalence and type of occupational injuries, (2) proximal factors predicting occupational injuries and (3) distal factors influencing occupational injuries.

#### The prevalence and type of injuries sustained

The study showed that more than half, (57.9%) of construction workers had experienced occupational injuries. The prevalence appears to be higher when compared to previous estimates elsewhere [32]. Even though there are no nationally representative data on the extent of injuries sustained by construction workers, the current prevalence is about 9.3 times higher than previous reports by the labour commission, which estimated that 6.2% of occupational accidents lead to deaths in construction [20]. Of relevance, the present finding demonstrates an increasing burden of injuries among workers. This confirms previous literature in Ghana and Europe which reported the industry to be injury-prone [17, 24, 25]. Specifically, injuries sustained by the workers were open wounds, superficial (on surface) and concussion and internal injury. These injuries are similar to the categories of injuries reported to be sustained by construction workers globally [33–38]. The study recommends that policymakers and occupational health experts should develop preventive and management strategies that incorporate specific injury part sustained. Also, future research should employ interventional studies to measure the effectiveness of preventive strategies for managing occupational injuries among construction workers. This could inform regulatory authorities to improve their health education and promotion campaigns.

#### Proximal factors predicting of occupational injuries

The proximal factors influencing occupational injuries highlight the immediate individual lifestyle characteristics (e.g. alcohol consumption, smoking, adherence to safety regulation, type of work and exposure to hazards) and socio-demographic profile. These factors had a relationship with occupational injuries. In this study, the socio-demographic profile such as age, sex, and income significantly predicted occupational injuries among construction workers. In particular, males were at high risk of being injured compared with females. In Ghana, the socio-cultural characteristics restrict the construction activities to males. This is due to the rigorous and hazardous nature of the construction industry [39]. Therefore, female workers are recommended to be placed in roles with limited risk in the industry. In addition, the income level of construction workers is a significant risk factor for occupation injury. Tasks with a high risk of injury usually require an expert to execute. However, in performing such duties, the worker’s exposure to such risk becomes higher which may eventually translate to injuries compared to other workers assigned to simple tasks. In Ghana, construction workers with this technical expertise are usually short in supply and also demand relatively higher wages than those doing menial and causal works. This possibly explains why workers in this category were at higher risk of occupational injury.

In addition, the risk of occupational injury increased with older workers compared to the youthful age group (economic active population). Older workers are susceptible to severe injury than younger ones [32, 40–42]. Aging process involves a series of physiological changes to the body that can make construction tasks very difficult for old people. The strength and ability required of a person to carry out physically demanding tasks effectively reduce as one age. Decrease cardiac output may affect a worker’s performance on physical demanding activity and increase his susceptibility to injury [43]. The study recommends stakeholders to consider the age profile, gender identity and socioeconomic status of construction workers when designing preventive and management strategies for injuries. In particular, future research should use a longitudinal approach to report a national representative data on occupational injuries among frontline construction workers. This can inform government policy and interventions. Further, researchers interested in occupational health-related issues should explore the reasons for the increased occupational injuries among different age, gender, and socio-economic wealth quintiles. For instance, future research should used a qualitative methods to explore the subjective experiences of frontline construction workers in managing occupational injuries.

#### Distal factors influencing occupational injuries

The distal factors predicting occupational injuries describe the organizational and work-related characteristics associated with the injuries. Specifically, the study showed that daily production targets, job location, work structure, trade specialization and off working days influence the risk of occupational injuries among frontline building construction workers. Working continuously without breaks, off-days and vacations predict the risk of injury among construction workers. Our study showed that a worker who enjoys off-days in a month is 96% protected from injury occurrence compared to those working every day throughout the month. Once a worker engages in continuous work without vacations, holidays and off-days, there is the possibility of fatigue setting in, and when this happens it affects performance and productivity [19]. Therefore, job schedules that offer workers limited opportunities for breaks, off-days, and vacations should be discouraged by managers of the industry.

Working on a permanent basis or as a temporal worker makes a worker more susceptibility to injuries more compared to working daily paid workers. Daily paid workers in the Ghanaian construction industry are normally engaged for relatively short periods, they visit job sites as and when they think they need to work. Such workers are mostly hired to carry out simple task which does not require any special expertise. Permanent workers are likely to experience the cumulative effects of the exposures on their health, unlike the daily paid workers. Finding from our study revealed that, workers operating from all locations and lower grounds were 71% and 39% protected from the possible risk of experiencing injury compared to working from a rooftop and elevated ground. This suggests that the chance of slipping from height and its impact on the individual is greater. This corroborates with previous findings from the US, UK, and South Africa which indicated that working from a height and elevation above the ground is the most cited cause of fall-related injuries [42, 44–46]. The study recommends that policymakers and occupational health experts should incorporate the identified distal factors in the planning and designing of construction projects as part of their injury prevention intervention as well as management strategies. We recommended that further research design with rigorous methods such as a cohort study should be employed to examine the causal relationship between the distal and proximate variables over time. Also, an interventional study is recommended for assessment of the effectiveness of organizational strategies that aim to improve the distal factors facilitating occupational injuries among frontline construction workers.

## Limitation

The study has limitations in terms of the scope, the target population, data-collection instrument, sampling, and possible recall bias. The study was limited to only construction workers in the Kumasi metropolis without incorporating the perspectives of regulatory bodies and workers in other parts of the country. Also, the instrument used to collect the data was a self-developed questionnaire based on previous literature, theory, and standard injury questionnaire. The study did not adopt any existing validated instrument. Nevertheless, several scientific scrutinies such as pre-testing of tools, random sampling, development of tools using previous literature, informed consent processes, statistical analysis, and discussion of findings in the context of relevant literature were employed to minimize biases.

## Data Availability

The datasets used and analyzed for this study are in the custody of the corresponding author and will be made available on a reasonable request.

## Abbreviations

AOR: adjusted odds ratio
CI: confident interval
GHS: Ghanaian cedis
ODK: Open Data Kit
OR: odds ratio
US $: United States dollar
